# Prospective evaluation of a complex public health intervention: lessons from an initial and follow-up cross-sectional survey of the tuberculosis strain typing service in England

**DOI:** 10.1186/1471-2458-14-1023

**Published:** 2014-10-02

**Authors:** Jessica Mears, Ibrahim Abubakar, Debbie Crisp, Helen Maguire, John A Innes, Mike Lilley, Joanne Lord, Ted Cohen, Martien W Borgdorff, Emilia Vynnycky, Timothy D McHugh, Pam Sonnenberg

**Affiliations:** Department of Infection and Population Health, University College London, London, UK; Centre for Infectious Disease Surveillance and Control, Public Health England, London, UK; George Elliot NHS Trust, Warwickshire, UK; Field Epidemiology Services Public Health England, London, UK; European Programme for Intervention Epidemiology, European Centre for Disease Control, Stockholm, Sweden; Department of Infection and Tropical Medicine, Birmingham Heartlands Hospital, Heart of England NHS Foundation Trust, Birmingham, UK; South Midlands and Hertfordshire Public Health England Centre, Hertfordshire, UK; Health Economics Research Group, Brunel University, London, UK; Division of Global Health Equity, Brigham and Women’s Hospital, Boston, USA; Department of Epidemiology, Harvard School of Public Health, Boston, Massachusetts USA; Department of Infectious Diseases, Public Health Service (GGD) Amsterdam, Amsterdam, The Netherlands; Department of Clinical Epidemiology, Academic Medical Centre, University of Amsterdam, Amsterdam, The Netherlands; Faculty of Epidemiology and Population Health, London School of Hygiene & Tropical Medicine, London, UK; Centre for Clinical Microbiology, Research Department of Infection, University College London, London, UK

**Keywords:** Tuberculosis, Strain typing, MIRU-VNTR, Complex intervention, Service evaluation

## Abstract

**Background:**

The national tuberculosis strain typing service (TB-STS) was introduced in England in 2010. The TB-STS involves MIRU-VNTR typing of isolates from all TB patients for the prospective identification, reporting and investigation of TB strain typing clusters. As part of a mixed-method evaluation, we report on a repeated cross-sectional survey to illustrate the challenges surrounding the evaluation of a complex national public health intervention.

**Methods:**

An online initial and follow-up questionnaire survey assessed the knowledge, attitudes and practices of public health staff, physicians and nurses working in TB control in November 2010 and March 2012. It included questions on the implementation, experience and uptake of the TB-STS. Participants that responded to both surveys were included in the analysis.

**Results:**

248 participants responded to the initial survey and 137 of these responded to the follow-up survey (56% retention).

**Knowledge**: A significant increase in knowledge was observed, including a rise in the proportion of respondents who had received training (28.6% to 67.9%, p = 0.003), and the self-rated knowledge of how to use strain typing had improved (‘no knowledge’ decreased from 43.2% to 27.4%).

**Attitudes**: The majority of respondents found strain typing useful; the proportion that reported strain typing to be useful was similar across the two surveys (95.7% to 94.7%, p = 0.67).

**Practices**: There were significant increases between the initial and follow-up surveys in the number of respondents who reported using strain typing (57.0% to 80.5%, p < 0.001) and the proportion of time health protection staff spent on investigating TB (2.74% to 7.08%, p = 0.04).

**Conclusions:**

Evaluation of a complex public health intervention is challenging. In this example, the immediate national roll-out of the TB-STS meant that a controlled survey design was not possible. This study informs the future development of the TB-STS by identifying the need for training to reach wider professional groups, and argues for its continuation based on service users’ perception that it is useful. By highlighting the importance of a well-defined sampling frame, collecting baseline information, and including all stakeholders, it provides lessons for the implementation of similar services in other countries and future evaluations of public health interventions.

**Electronic supplementary material:**

The online version of this article (doi:10.1186/1471-2458-14-1023) contains supplementary material, which is available to authorized users.

## Background

Complex public health interventions – interventions involving multiple interacting components – when applied at a national level, are often implemented in a way that makes evaluating them with rigorously designed trials difficult [1]. Instead, they require a more pragmatic approach using the available data [2].

Molecular typing of *Mycobacterium tuberculosis* is a tool for TB surveillance and control. It has been used in combination with epidemiological information to identify outbreaks [3], identify new routes of transmission [4], refute suspected transmission [5, 6], evaluate TB control programmes [7, 8] and detect laboratory cross contamination [9, 10].

The National Tuberculosis Strain Typing Service (TB-STS) is a complex public health intervention involving laboratory, public health and clinical services across England and was introduced in January 2010 [11]. A mixed-method prospective evaluation of the acceptability, implementation, effectiveness and cost-effectiveness of the service was undertaken [12]. Here we report in detail on one component of the evaluation: a cross-sectional initial and follow-up survey of those delivering and using the TB-STS to assess their knowledge, and to understand the impact of the service on changes in attitudes and practices associated with strain typing.

## Methods

### Intervention

A full description of the TB-STS, with laboratory guidelines for MIRU-VNTR strain typing and reporting [13] and a handbook for public health actions relating to cluster investigations (TB strain typing and cluster investigation handbook [14]) can be found on the Health Protection Agency website [11]. Briefly, the TB-STS involves prospectively typing the first *M. tuberculosis* isolate from every culture-confirmed tuberculosis (TB) patient using 24 locus Mycobacterial Interspersed Repetitive Units-Variable Number Tandem Repeats (MIRU-VNTR), a standardised molecular typing method [15]. Based on the strain type result, patients are grouped into ‘clusters’ [13, 14] which are reported to the Health Protection Units (HPUs). If a cluster meets a certain threshold, as outlined in the TB strain typing and cluster investigation handbook, [14] then a cluster investigation is launched to try to establish epidemiological links between the clustered patients, thereby identifying the transmission setting and/or an outbreak. As part of a cluster investigation the HPU may decide to carry out enhanced contact tracing or screening around the patients in the cluster or the identified transmission setting. By combining patients’ strain type with epidemiological information the TB-STS aims to inform public health decision-making at the local level.

The various components of the TB-STS were implemented at different times (but always on a national scale): prospective strain typing was introduced across England in January 2010; one cluster investigator was appointed in January 2010 and the remaining two were appointed in January 2011; the training programme for health protection staff working in HPUs was carried out between January 2011 and February 2012, consisting of a seminar at the national Health Protection Conference, an online seminar, a workshop conducted at each HPU, the publication of the handbook [14] and a Q&A sheet [11] (in December 2010); and the software linking patients’ electronic TB record and strain typing result with information from clusters investigations was not developed during this study period.

### Study design

An initial survey was conducted in November 2010 and a follow-up survey in March 2012 using a web-based survey questionnaire ( http://www.objectplanet.com/opinio). The target population were all public health staff, chest/respiratory physicians and TB nurses working in TB control in England. Questions were asked about the knowledge (awareness of the service, training, resources and self-reported knowledge), attitudes (perceived usefulness of the service) and practice (if and how strain typing is accessed and used, and its associated workload). All questions and possible responses are available in the appendix (Additional file 1). The survey was piloted with a nurse, a physician and a public health specialist. The initial survey was emailed to all users of the TB notification system [16] and to staff responsible for TB control in HPUs who were asked to pass it on to their local TB teams; the sampling frame could not be enumerated. The follow-up survey was emailed to respondents to the initial survey.

### Analysis

Participants that responded to both surveys were included in the statistical analysis. Responses from people working at national, regional or PCT-level, including cluster investigators, and people working in Wales were excluded from this analysis. We compared the knowledge, attitudes and practices of public health and clinical staff working on TB control in the initial and follow-up surveys by calculating and comparing medians and inter-quartile ranges (IQR), and means and standard deviations (SD), and using two-sample t-tests, chi^2^ tests and logistic regression, where appropriate. Calculations exclude item non-responses. Analyses were conducted overall, by professional category and the TB incidence of the HPU area in which respondents worked (low, medium and high incidence defined as an annual notification rate of <10/100,000 population, 10 to 19/100,000 and ≥20/100,000 respectively).

### Ethical considerations

The study was classified as a service evaluation by University College London Hospital Foundation Trust therefore specific ethical approval was not required.

## Results

### Survey responses

**Figure 1:**
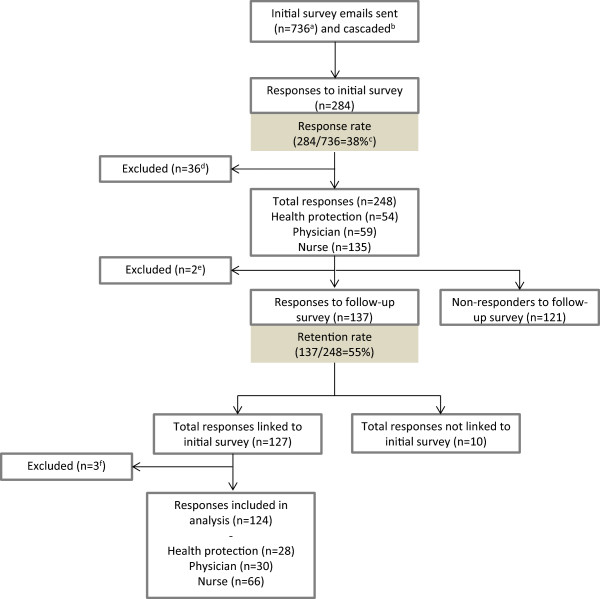
**Responses to the initial and follow-up surveys.** ^a^The email was sent to all users of the Enhanced TB Surveillance database. This included all administrative staff as well as well as staff working at national, regional and Primary Care Trust level, for whom the survey may not be relevant. ^b^It is not known how many people received the email via through the HPU cascade. ^c^This response rate is an underestimation because of the denominator used. ^d^Respondents working at national, regional or PCT-level (n = 27) and those from Wales (n = 9) were excluded from this analysis. ^e^Email addresses not available from the initial survey (n = 2). ^f^In some cases it was not possible to link the follow-up response to the initial response (n = 10). Respondents working at national, regional or PCT-level and those from Wales (n = 4) were excluded from this analysis.

There were 248 responses to the initial survey, 137 responses to the follow-up survey (55% retention), and for 124 we have responses to both the initial and follow-up surveys (Figure 1). Respondents to the initial survey who did not respond to the follow-up survey were not significantly different to those that responded to both: no particular profession, full-time/part-time position or those working in areas with different TB incidences was more (or less) likely to respond to the follow-up survey, and there was no significant difference between the proportion of people who had heard of the TB-STS or had access to strain typing at the time of the initial survey (Table 1). Respondents were from all nine regions of England and covered 24 (of 26) HPUs.

**Table 1:** **Characteristics of responders and non-responders to the follow-up survey**

		Initial and follow-up responses ^a^		Non-responders to the follow-up survey	
		N	%	n	%
Total		124		121	
Profession	HPU	28	22.6	23	19.0
	Physician	30	24.2	29	24.0
	Nurse	66	53.2	69	57.0
TB incidence^b^	Low	56	45.2	50	42.0
	Medium	33	26.6	32	26.9
	High	35	28.2	37	31.1
Work time	Full-time	95	79.2	87	77.0
	Part-time	25	20.2	26	21.5
Heard of the TB-STS		105	85.4	100	84.7
Access to strain typing		90	72.6	99	81.8

^a^Using the information reported in the initial survey.

^b^Area where respondents worked is defined as low, medium and high TB incidence: <10/100,000, 10-19/100,000, ≥20/100,000 population, respectively.

There were no significant differences between characteristics of non-responders and responders, including access to strain typing (81.8 % vs. 72.6 %, chi^2^ test p = 0.085).

### Knowledge

**Figure 2:**
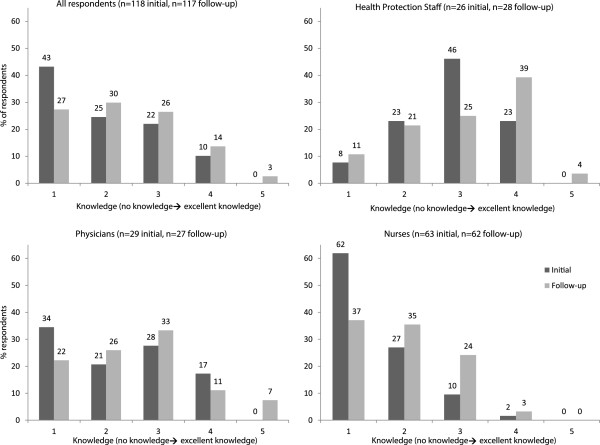
**Self-reported knowledge of strain typing.** Self-reported knowledge about how to use strain typing was scored on a scale of 1 to 5, where 1 represented ‘no knowledge’ and 5 represented ‘excellent knowledge’. Dark bars represent responses to the initial survey and light bars represent responses to the follow-up survey.

Between the initial and follow-up surveys there were increases in the proportion of respondents who had heard of the TB-STS, had access to strain typing results, had received training, and had access to training resources (Table 2). The self-rated knowledge of how to use strain typing also increased over time (Figure 2). Nurses reported lower average knowledge in both surveys compared to physicians and health protection staff.

**Table 2:** **Knowledge: Awareness to the TB-STS and access to strain typing data and resources**

		Initial survey		Follow-up survey		
		n	%	n	%	p-value ^d^
**Heard of the TB-STS** ^**a**^	Total	105	85.4	123	99.2	<0.001
Profession	Health protection	28	100	28	100	.
	Physician	20	66.7	30	100	0.001
	Nurse	57	86.4	65	98.5	0.015
TB incidence	Low	49	87.5	56	100	0.006
	Medium	24	72.7	32	97.0	0.010
	High	32	91.4	35	100	0.077
**Access to strain typing data** ^**b**^	Total	90	72.6	108	87.1	0.004
Profession	Health protection	26	92.9	27	96.4	0.553
	Physician	21	70.0	23	76.7	0.559
	Nurse	43	65.2	58	87.9	0.002
TB incidence^c^	Low	38	67.9	47	83.9	0.047
	Medium	24	72.7	28	84.9	0.228
	High	28	80.0	33	94.3	0.074
Access to training	(health protection staff)	8	28.6	19	67.9	0.003
Access to resources	(health protection staff)	16	57.1	23	82.1	0.042

^a^Have you heard of the TB-STS (apart from in this survey)? *(Yes / No).*

^b^Do you have access to strain typing data? *(Yes / No).*

^c^Area where respondents worked is defined as low, medium and high TB incidence: <10/100,000, 10-19/100,000, ≥20/100,000 population, respectively.

^d^chi^2^ test of significance comparing responses from the initial and follow-up surveys.

### Attitudes

69 people (69/124 = 56%) from the initial survey and 95 people (95/124 = 77%) from the follow-up survey reported that they used strain typing. Opinions of the usefulness of TB strain typing was high amongst all respondents and did not change between the surveys (95.7% to 94.7%, p = 0.667; Table  3). A greater proportion of respondents from low TB incidence areas found strain typing useful, compared to those working in high TB incidence areas (97.4% compared to 89.3% in the follow-up survey, respectively), though this result was not statistically significant (OR = 0.13, 95% CI 0.014-1.128, p = 0.075).

**Table 3:** **Attitudes: Number and proportion of respondents that reported strain typing to be useful** ^**a**^

		Initial survey				Follow-up survey ^b^				
		Useful		Not useful		Useful		Not useful		
		n	%	n	%	n	%	n	%	P ^d^
Total respondents that reported using strain typing		66	95.7	3	4.3	89	94.7	5	5.3	0.667
Profession	Health protection	22	95.7	1	4.3	24	96.0	1	4.0	0.952
	Physician	16	100	0	0.0	20	95.2	1	4.8	0.464
	Nurse	28	93.3	2	6.7	45	93.8	3	6.3	0.942
TB incidence^c^	Low	31	100	0	0.0	38	97.4	1	2.6	0.450
	Medium	16	94.1	1	5.9	26	96.3	1	3.7	0.736
	High	19	90.5	2	9.5	25	89.3	3	10.7	0.892

^a^The following question was asked to respondents who reported that they used strain typing data for TB control (Figure 3): Do you find the strain typing information useful? *(Very useful / Quite useful / Not very useful / Useless)* ‘Very useful’ and ‘Quite useful’ are grouped into ‘useful’, and ‘Not very useful’ is presented as ‘Not useful’. No one reported finding the strain typing ‘useless’ in either survey.

^b^One response was missing from the follow-up survey.

^c^Area where respondents worked is defined as low, medium and high TB incidence: <10/100,000, 10-19/100,000, ≥20/100,000 population, respectively.

^d^chi^2^ test for significance comparing responses from the initial and follow-up surveys, missing items were excluded.

### Practices

**Figure 3:**
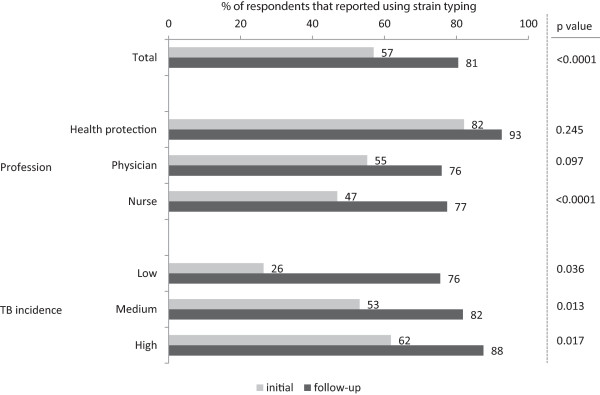
**Practices: Respondents that use strain typing for TB control** ^**a**^ **.** The proportion respondents that reported using strain typing for TB control. Dark bars represent responses to the initial survey and light bars represent responses to the follow-up survey. P-values from chi^2^ tests for significance comparing initial and follow-up proportions are shown. ^a^How often do you use strain typing data in your case management of outbreak investigation? *Never / For few cases / For about half of cases / For many cases / For every case*. ‘For few cases’, ‘for about half of cases’, ‘for many cases’ and ‘for every case’ were grouped to show the proportion of respondents that use strain typing.

Figure 3 shows a significant increase in the number of respondents that reported using strain typing between the initial and follow-up surveys. There was an increase in the number of respondents who reported using strain typing to identify links between cases (65.3% to 78.2%, p = 0.02; the most common use), disprove links between cases (46.8% to 58.9%, p = 0.06) and to justify stopping contact tracing (20.2% to 30.7%, p = 0.06) (Table 4).

**Table 4:** **Practices: How respondents use strain typing data** ^**a**^

			Initial survey		Follow-up survey		
			n	%	n	%	P ^c^
**Identify clusters and links between cases**			81	65.3	97	78.2	0.024
	Profession	Health protection	22	78.6	25	89.3	0.275
		Physician	18	60.0	21	70.0	0.417
		Nurse	41	62.1	51	77.3	0.058
	TB incidence^b^	Low	34	60.7	41	73.2	0.160
		Medium	20	60.6	28	84.8	0.027
		High	27	81.8	28	84.8	0.771
**Disprove clusters and links between cases**			58	46.8	73	58.9	0.056
	Profession	Health protection	21	75.0	24	85.7	0.313
		Physician	13	43.3	15	50.0	0.605
		Nurse	24	36.4	34	51.5	0.079
	TB incidence^b^	Low	27	48.2	33	58.9	0.256
		Medium	15	45.5	22	66.7	0.083
		High	16	48.5	18	54.5	0.632
**Justify extended contact tracing**			51	41.1	60	48.4	0.250
	Profession	Health protection	16	57.1	19	67.9	0.408
		Physician	11	36.7	10	33.3	0.787
		Nurse	24	36.4	31	47.0	0.217
	TB incidence^b^	Low	20	35.7	25	44.6	0.335
		Medium	13	39.4	19	57.6	0.139
		High	18	54.5	16	48.5	0.632
**Justify stopping contact tracing**			25	20.2	38	30.6	0.058
	Profession	Health protection	13	46.4	13	46.4	1
		Physician	3	10.0	5	16.7	0.448
		Nurse	9	13.6	20	30.3	0.021
	TB incidence^b^	Low	9	16.1	18	32.1	0.047
		Medium	8	24.2	13	39.4	0.186
		High	8	24.2	7	21.2	0.771
**To provide more information**			34	27.4	44	35.5	0.171
	Profession	Health protection	13	46.4	10	35.7	0.415
		Physician	5	16.7	6	20.0	0.739
		Nurse	16	24.2	28	42.4	0.027
	TB incidence^b^	Low	15	26.8	19	33.9	0.411
		Medium	8	24.2	12	36.4	0.284
		High	11	33.3	13	39.4	0.615

^a^What do you use strain typing for? (multiple selections possible) (*Don’t know / Identify clusters and links between cases / Disprove clusters and links between cases / Justify extended contact tracing / Justify stopping contact tracing / To provide more information / Other (please specify)).*

^b^Area where respondents worked is defined as low, medium and high TB incidence: <10/100,000, 10-19/100,000, ≥20/100,000 population, respectively.

^c^chi^2^ test for significance comparing responses from the initial and follow-up surveys.

Table 5 shows workload reported by nurses and health protection staff. For the nurses, no significant changes in contact tracing workload were reported.

**Table 5:** **Practices: the workload associated with the TB-STS for nurses and health protection staff**

		TB incidence ^a^	Survey	n ^b^	median	(IQR)	mean	(SD)	p-value ^c^
Nurses	No. contacts screened in the last month	Total	Initial	57	21	(11–36)	37.1	(53.5)	
			Follow-up	55	20	(8–40)	33.9	(45.1)	0.37
		Low	Initial	26	16	(6–35)	23.8	(24.8)	
			Follow-up	23	15	(6–25)	17.2	(13.8)	0.13
		Medium	Initial	17	25	(14–30)	30.2	(26.2)	
			Follow-up	18	23	(15–42)	43.7	(43.7)	0.18
		High	Initial	14	32.5	(14–100)	70.2	(93.3)	
			Follow-up	14	16.5	(10–80)	48.6	(58.9)	0.24
	No. hours spent on contact tracing in the last month	Total	Initial	55	8	(4–16)	12.0	(10.8)	
			Follow-up	52	7.5	(3.5-15.5)	16.1	(41.7)	0.24
		Low	Initial	25	8	(3–14)	10.1	(10.5)	
			Follow-up	21	6	(3–15)	11.5	(14.7)	0.35
		Medium	Initial	16	12	(4–23)	14.4	(11.4)	
			Follow-up	18	7.5	(4–12)	10.2	(7.8)	0.10
		High	Initial	14	9	(6–15)	12.5	(10.8)	
			Follow-up	13	8	(3–16)	31.9	(81.1)	0.19
	% time spent on contact tracing	Total	Initial	57	20	(10–30)	24.2	(16.5)	
			Follow-up	54	20	(10–25)	21.7	(17.6)	0.22
		Low	Initial	26	20	(10–25)	21.2	(16.1)	
			Follow-up	23	20	(6–25)	21.8	(19.5)	0.45
		Medium	Initial	17	20	(20–30)	24.1	(13.8)	
			Follow-up	17	20	(10–25)	19.4	(10.4)	0.14
		High	Initial	14	30	(15–40)	30.0	(19.7)	
			Follow-up	14	20	(10–40)	24.4	(21.7)	0.24
Health protection staff	Investigations initiated because of epidemiological links	Total	Initial	23	0	(0–1)	0.5	(0.8)	
			Follow-up	21	1	(0–2)	2.8	(6.1)	0.04
		Low	Initial	15	0	(0–1)	0.3	(0.62)	
			Follow-up	14	0.5	(0–1)	1.5	(2.3)	0.04
		Medium	Initial	3	1	(0–1)	0.7	(0.7)	
			Follow-up	3	1	(1–4)	2.0	(1.7)	0.14
		High	Initial	5	0	(0–1)	0.8	(1.3)	
			Follow-up	4	1.5	(0.5-15)	7.8	(13.5)	0.14
	Strain typing used to provide more information in epidemiological investigation	Total	Initial	22	0	(0–1)	0.6	(1)	
			Follow-up	22	1	(0–2)	1.8	(2.5)	0.03
		Low	Initial	14	0	(0–1)	0.4	(0.8)	
			Follow-up	14	0.5	(0–2)	1.4	(2)	0.05
		Medium	Initial	4	0.5	(0–2)	1.0	(1.4)	
			Follow-up	3	1	(0–2)	1.0	(1)	0.50
		High	Initial	4	0.5	(0–2)	1.0	(1.4)	
			Follow-up	5	2	(1–3)	3.2	(4)	0.17
	Strain typing influences an epidemiological investigation	Total	Initial	23	0	(0–1)	0.8	(1.1)	
			Follow-up	14	0.5	(0–2)	1.2	(1.6)	0.17
		Low	Initial	14	0	(0–1)	0.4	(0.8)	
			Follow-up	8	0	(0–0.5)	0.6	(1.4)	0.34
		Medium	Initial	4	0.5	(0–2)	1.0	(1.4)	
			Follow-up	2	1	(0–2)	1.0	(1.4)	0.50
		High	Initial	5	1	(1–3)	1.6	(1.3)	
			Follow-up	4	2	(1.5-3.5)	2.5	(1.7)	0.20
	Investigation initiated because of strain typing	Total	Initial	23	0	(0–2)	2.2	(6.3)	
			Follow-up	22	0	(0–1)	1.1	(2.3)	0.79
		Low	Initial	14	0	(0–1)	0.4	(0.8)	
			Follow-up	14	0	(0–0)	0.5	(1.3)	0.43
		Medium	Initial	4	0.5	(0–1)	0.5	(0.6)	
			Follow-up	4	0.5	(0–1.5)	0.8	(1)	0.34
		High	Initial	5	4	(3–6)	8.6	(12.2)	
			Follow-up	4	1	(1–5.5)	3.3	(4.5)	0.78
	Epidemiological links identified in strain typing cluster	Total	Initial	22	0	(0–0)	0.4	(0.8)	
			Follow-up	20	0	(0–0)	0.4	(0.8)	0.52
		Low	Initial	13	0	(0–0)	0.2	(0.6)	
			Follow-up	13	0	(0–0)	0.3	(0.9)	0.30
		Medium	Initial	3	0	(0–1)	0.3	(0.6)	
			Follow-up	3	0	(0–0)	0.0	(0)	0.81
		High	Initial	6	0.5	(0–1)	0.8	(1.2)	
			Follow-up	4	0.5	(0–1.5)	0.8	(1)	0.55
	% time spent on investigations	Total	Initial	23	1	(0–5)	2.7	(3.2)	
			Follow-up	25	5	(0–5)	7.2	(11.1)	0.04
		Low	Initial	15	0	(0–5)	2.1	(3.1)	
			Follow-up	15	5	(0–12)	8.3	(13.1)	0.04
		Medium	Initial	3	5	(0–5)	3.3	(2.9)	
			Follow-up	4	5	(2.5-5)	3.8	(2.5)	0.42
		High	Initial	5	5	(1–5)	4.4	(3.7)	
			Follow-up	6	3.5	(0–5)	6.2	(9.5)	0.35

^a^Area where respondents worked is defined as low, medium and high TB incidence: <10/100,000, 10-19/100,000, ≥20/100,000 population, respectively.

^b^n is number of people who answered the question.

^c^Paired t-test comparing initial and follow-up responses.

Health protection staff reported a significant increase in the mean number of investigations initiated because of epidemiological links between patients over a three month period (mean 0.5 to 2.8, p = 0.04) and the mean number of these investigations for which strain typing was used to provide more information (0.6 to 1.8, p = 0.03), but there was no change in the number that were influenced by the strain typing (1.2 to 0.4, p = 0.17). There was no reported difference in the number of clusters investigated because of their strain type (in high incidence areas a large, but non-significant, decrease was reported) and the number of strain typing investigations that identified epidemiological links between cases remained low (Table 5). Overall, the proportion of time health protection staff spent on cluster investigations increased significantly (from 2.7% to 7.2%, p = 0.04).

There was no reported change over time in the frequency at which physicians were called to incident meetings (a meeting, often multi-disciplinary, held to discuss a TB patient, group or cluster of cases that are of particular concern) (p = 0.503; most reported once every three months or less (65.5% at in the initial survey and 67.9% at follow-up)) and there was no change in the number of physicians who reported strain typing as being relevant to an incident meeting (57.8% to 55.6%, p = 0.875).

## Discussion

### Main findings

We present results from an initial and follow-up survey assessing the knowledge, attitudes and practices of those implementing and using the TB-STS. There were 124 responses to both the surveys, representing health protection staff and clinic-based physicians and nurses from 24 (of 26) HPUs across England. Strain typing was used by more people, and an increase in knowledge of the TB-STS was reported at the follow-up survey. A change in attitude was not measured as the majority of respondents found strain typing useful to them at both time points. With respect to workload associated with the TB-STS, there was no change over time in the contact tracing activities of nurses or the frequency of incident meetings attended by physicians; however the proportion of time health protection staff spent on investigating TB transmission increased significantly. Despite strain typing being used to provide more information to public health staff at follow-up, there was no increase in epidemiological links identified.

### How this relates to previous studies

National TB strain typing services have been introduced in other countries [17–20], but the knowledge, attitudes and practices of users have not been evaluated. However, the impact of strain typing on contact tracing activities in the Netherlands has been assessed [5]. Consistent with this study, we found no change in the workload associated with strain typing for nurses and physicians, even though strain typing was used by more people at the follow-up survey (indicating the successful roll-out of the service). This may be because it is difficult to measure marginal changes in workload associated with a particular service where the workforce is already working at full capacity. Health protection staff, however, spent a greater proportion of time on cluster investigations. Given that the Handbook had not been published and all the cluster investigation coordinators were not in position at the time of the initial survey, this is not surprising and suggests that the TB-STS had been integrated into the TB control activities of the HPUs.

Based on evidence from the USA one would expect more possible transmission links to be identified when strain typing informs contact tracing activities [6, 8]. However, in this study we found the proportion of time health protection staff spent on cluster investigations increased and the number of investigations that used strain typing increased, but there was no increase in the reported number of possible transmission links found between clustered cases. This discordance between the findings on subjective report of utility and the public health outcomes reported could be because the current methods used by public health staff to identify epidemiological links may be inappropriate or ineffective, or there may have been an increase in suspected (but not established) transmission because of the strain typing information. For the TB-STS to have a public health impact and reduce TB transmission, cluster investigations would have to lead to the detection of previously unidentified latently infected and active TB cases.

### Limitations

The way the TB-STS was implemented and the survey design have resulted in a number of limitations that provide important lessons for the TB-STS, the evaluation of future services and other complex interventions.

Firstly, the survey was developed after the initiation of the TB-STS so baseline information could not be collected and we are likely to have underestimated the difference between the surveys. However, the initial survey was conducted before the roll-out of any training for the TB-STS and prior to the employment of all national staff to coordinate cluster investigations. An alternative study design, which would have had a control group, would have been possible if the TB-STS was rolled out in a step-wise process across the country, rather than nationally.

Secondly, the target population for the survey was all public health staff, physicians and nurses working in TB control in England. It was not possible to enumerate the sampling frame because no formal or informal register of clinical and health protection staff working in TB could be identified. As a result, we could not calculate a response rate.

Finally, the 50% retention rate between the surveys is quite low and we may have lost the opinions and experiences of a particular group of people. However, non-responders to the follow-up survey did not differ significantly to those that responded to both surveys based profession or burden of TB in their geographical area. Because the study was conducted as part of a programmatic service implementation, results must be interpreted accordingly.

### Recommendations

The findings of this survey inform the development of the TB-STS and the design of future evaluations. Despite a significant increase in the number of health protection staff who had received training, there remained some that had not received any, suggesting the need for an ongoing training programme that also takes into account turnover of staff. Self-reported knowledge of how to use the strain typing information was lower for nurses compared with physicians and health protection staff, possibly representing a gap in the training strategy, which did not include nurses. The finding that physicians had the highest self-reported knowledge across the two surveys, even though they were not included in the training strategy, might be because they have had access to information on strain typing from other sources and, relative to nurses and public health staff, might self-rate their knowledge higher.

The perception of usefulness did not change over time as most people found strain typing to be useful in both surveys. This suggests that any changes in practice are due to increasing knowledge and access to strain typing, rather than attitudes towards strain typing. Therefore, to improve use and impact of the TB-STS, there should be a focus on improving training and making strain typing data easily accessible so that it can become better integrated into the TB service.

The findings of this survey argue for the continuation of the TB-STS. A majority of people reported the TB-STS to be useful and health protection staff reported an increase in the number of investigations for which strain typing was used to provide more information, although there was no increase in the number of investigations that were influenced by strain typing. This discordance between the findings on subjective report of utility and the investigation outcomes reported may signify the high value placed on information.

When implementing a public health intervention and planning an evaluation it is essential to have a well-defined sampling frame and a baseline that can be measured before the start of the service implementation. Where possible, the evaluation of a service should start prior to its implementation in order to capture the baseline and to design the evaluation based on the planned service implementation. This survey is an example of where this was not possible and highlights the importance of acknowledging the context in which the service was implemented, both for assessing its success and understanding the limitations of the evaluation design.

The variation in knowledge, attitudes and practices across the professions illustrates the importance of including all the service stakeholders in the evaluation. For example, in the TB-STS, nurse respondents reported lower knowledge, suggesting that they could benefit from being included in the training strategy.

This survey is the first component of the evaluation of the TB-STS. To better understand the public health utility and evaluate the impact of such a service, a comprehensive mixed-methods evaluation is underway [12]. This includes modelling of the effectiveness and cost-effectiveness and qualitative studies.

## Conclusions

Evaluating a complex public health intervention requires a pragmatic approach, taking into account how the service has been implemented. In these initial and follow-up surveys, public health staff, physicians and TB nurses found the TB-STS useful and increased the amount they used it in the first two years of the service, arguing for the continuation of the service. Despite this, the impact of the TB-STS on cluster investigations remained unclear. We recommend continuing the service but with ongoing and more thorough training of service users and focussing on improving knowledge and making data more accessible. Future evaluations of complex interventions should be initiated prior to the implementation of the service, and would benefit from an enumerable sampling frame and a measurable baseline.

## Electronic supplementary material

Additional file 1: **Survey Questions.** (PDF 310 KB)

## Abbreviations

HPU: Health Protection Unit
MIRU-VNTR: Mycobacterial interspersed repetitive units-variable number tandem repeats
NHS: National Health Service
PHE: Public Health England
TB: Tuberculosis
TB-STS: Tuberculosis strain typing service.
