# Supplementary material for: Prospective evaluation of a complex public health intervention: lessons from an initial and follow-up cross-sectional survey of the tuberculosis strain typing service in England
Source: BMC Public Health. 2014 Oct 2;14:1023. doi: 10.1186/1471-2458-14-1023 (PMC4194411; doi:10.1186/1471-2458-14-1023)
Supplement: Supplementary file 1 — Additional file 1: Survey Questions. (PDF 310 KB) [file 12889_2014_7131_MOESM1_ESM.pdf]

Survey Questions

Questions that are numbered without a letter were asked to all participants. Where a letter follows the question number the question was asked to that particular group (x=nurses, y=physicians, z=public health staff). The questions are divided by target group for Sections C and D.

--

TB Control and Strain Typing: A Questionnaire for Nurses / Physicians / HPU staff

The Health Protection Agency has commissioned an evaluation of the National TB Strain Typing Service (STS). We are interested in how much time nurses / physicians / staff from HPUs spend on cluster investigations and their uptake and experience of using strain typing data. This is the second part of a two-part questionnaire (the first part was conducted in November 2010). All the information collected in this form will be anonymised and stored according to the Data Protection Act; we do ask for your name and contact details so we can link this to your response - oryour predecessor's response - to the first part of the questionnaire.

THANK YOU FOR AGREEING TO TAKE PART IN THIS QUESTIONNAIRE

You can save the questionnaire and return to it another time by clicking "save" at the bottom of any page and following the instructions.

If you have any queries please do not hesitate to contact Jessica Mears, evaluation scientist - jessica.mears@hpa.org.uk, 0208 327 6265

Section A -- This section asks basic information about where you work

1. Name
2. Email address
3. Telephone number
4. Job title
5.
    x. Which Health Protection Unit does your workplace fall under? (multiple selections available) *List of 26 HPUs*
    y. Which Health Protection Unit does your workplace fall under? (multiple selections available) *List of 26 HPUs*
    z. Do you work at the national level, regional level, or Health Protection Unit level (or other)? *National / Regional / HPU / other*

Section B -- The following questions are about your current awareness and use of TB strain typing

1. Have you heard of the National TB Strain Typing Service (aside from this questionnaire)? *Y/N*
2. How do you access strain typing data? (multiple selections possible) *Don't get data / Send request for specific cases / Automatically receive for each case / Contact labs directly / Cluster reports from lab / Cluster report/phone call from HPU / Web-based ETS/LTBR / Contacted by a cluster investigator / Other*

*If you have answered "*Don't get data*" in Section B Q2, skip to BQ7

3.
    x. - How many weeks after a sample is sent to the lab do you receive the individual strain type? (weeks)
    y. - How many weeks after a sample is sent to the lab do you receive the individual strain type? (weeks)
    z. Do you usually access/get hold of strain typing data before or after you have finished contact tracing? *Before / After*
4. What do you use strain typing for? (multiple selections possible) *Don't know / Identify clusters and links between cases / Disprove clusters and links between cases / Justify extended contact tracing / Justify stopping contact tracing / To provide more information / Other (please specify)*
5. How often do you use strain typing data in your case management or outbreak investigation? *Never / For few cases / For about half of cases / For many cases / For every case*
   Any comments you have about how often you use the strain typing data would be helpful
6. Do you find the strain typing information useful? *Very useful / Quite useful / Not very useful / Useless*
   Any comments you have about how useful you find the strain typing data would be helpful

*Skip to BQ8

7. If you had access to strain typing data, what would you use it for? (multiple selections possible) *Don't know / Identify clusters and links between cases / Disprove clusters and links between cases / Justify extended contact tracing / Justify stopping contact tracing / To provide more information / Other (please specify)*

8. Have you received any training on using strain typing data? *Yes (please specify) / No*
9. Do you have access to any resources or tools to help interpret and use strain typing information? (multiple selections possible) *No / Handbook / Training workshops / Colleagues / Webcast/online training / Other (please specify)*
10. How would you rate your knowledge of how to interpret MIRU-VNTR data? *(no knowledge) 1 / 2 / 3 / 4 / 5 (Excellent knowledge)*

Section C -- The following questions are about your current TB-related workload

CX: Questions for Nurses

1. What are your working hours? *Full time / Part time / Other (please specify)*
2. Do you work on anything other than TB? *Yes / No*

*If you have answered "No" in Section CX Q2, skip to CXQ4

3. What proportion of your average week do you spend on TB-related work? *%*
4. Are you involved in contact tracing? *Yes / No*

*If you have answered "No" to CXQ4, skip to Section DX

We are now going to ask a few questions about contact tracing and the <u>contacts</u> of TB patients.

5. How do you do your contact tracing work? *Dedicated time in clinic (% of contact tracing done in this way) / Telephone interviews (%) / Home visit (%) / Other (please specify)*
6. In total, how many contacts have you screened in the last month? (using any of these methods: Mantoux, X-ray, IGRA, symptom check) (Number of people)
7. How many hours did this take? (Hours)
8. In the last month, what proportion (%) of your total working time have you spent on tracing and screening contacts in and out of clinic? (% of time)

We are now going to ask you some questions about <u>newly diagnosed TB patients.</u>

9. How many new active TB cases have you managed in the last full month? (cases)
10. How many of these required contact tracing? (cases)

This section asks you about <u>5 recent index cases</u> for whom contact tracing has been completed. It might help to have your case management notes in front of you before starting this section.

11. Please fill in the following table based on 5 recent index cases whose contacts have been screened:

	Case 1	Case 2	Case 3	Case 4	Case5
ETS / LTBR No.					
Age / year of birth					
Site of disease	*Pulmonary / extra-pulm*	*Pulmonary / extra-pulm*	*Pulmonary / extra-pulm*	*Pulmonary / extra-pulm*	*Pulmonary / extra-pulm*
Smear test result	*Positive / Negative / Unknown*	*Positive / Negative / Unknown*	*Positive / Negative / Unknown*	*Positive / Negative / Unknown*	*Positive / Negative / Unknown*
Culture result	*Positive / Negative / Unknown*	*Positive / Negative / Unknown*	*Positive / Negative / Unknown*	*Positive / Negative / Unknown*	*Positive / Negative / Unknown*
When was the strain type available to you?	*Before contact investigation / During contact investigation / After contact investigation / Never*	*Before contact investigation / During contact investigation / After contact investigation / Never*	*Before contact investigation / During contact investigation / After contact investigation / Never*	*Before contact investigation / During contact investigation / After contact investigation / Never*	*Before contact investigation / During contact investigation / After contact investigation / Never*
No. contacts identified					
No. contacts screened					
No. contacts with active TB disease					
No. contacts with latent TB infection					

CY: Questions for Physicians

1. What are your working hours? *Full time / Part time / Other (please specify)*
2. What kind of physician are you?
3. What proportion of your average week do you spend on TB-related work? (%)
4. On average throughout the year, how many new TB cases do you personally start on treatment per month? (cases)
5. How often are you called to an incidence meeting to discuss contact tracing/screening? *Once a month or more / Once every 1-3 months / Once every 3-6 months / Less than once every 6 months / Never*
6. Has the strain type of the case under discussion ever been relevant in an incident meeting? *Yes / No*
   If yes, please could you give some more details about the incident(s)

CZ: Questions for Public Health Staff

1. What are your working hours? *Full time / Part time / Other (please specify)*
2. Do you work on anything other than TB? *Yes / No*

*If you answered “No” to Section CZ Q2 skip to CZQ5

3. What proportion of your average week do you spend on TB-related work? (%)
4. On average, how many hours per week do you spend on TB-related work? (Hours)
5. In the last 3 months, how many TB cases have been reported in your workplace (HPU, region or nationally)? (cases)

6. In the last 3 months, how many extended contact investigations were initiated because the cases were known contacts (regardless of strain type)? (extended contact investigations)
7. In how many of these was strain typing used to provide more information?
8. In how many of these investigations – investigations that were initially opened because cases were known contacts of each other – did strain typing influence your investigation? *Number of investigations lengthened or reopened / Number of investigations stopped*
   If possible, please provide more information about these incidents.

9. In the last 3 months, how many cluster investigations were initiated because of strain typing data (i.e. where links between cases were not identified through initial contact tracing)? (cluster investigations)
10. In how many of these investigations that were initiated because of the strain typing data were epidemiological links found between cases following the cluster investigation?
    If possible, please provide more information about these incidents.

11. In the last month, what proportion (%) of your time have you spent on cluster investigation activities (investigating epidemiologically linked or strain type-linked cases)? (%)
12. If you have any other comments about how you use (or don't use) strain typing data and how useful you find it please write in the box below.

Section D -- The following questions are about investigating transmission

DX: Questions for Nurses

In the last year have you investigated any clusters of 2 or more TB cases because…

1. …they were contacts of each other (not because of strain type)? *Yes / No*
   Comments welcome
2. …they were linked by strain type along? *Yes / No*
   Comments welcome

3. In the last year have you stopped investigating possible transmission because the cases involved had different strain types? *Yes / No*
   If yes, please could you give some more details about the incident.

4. If you have any other comments about how you use (or don't use) strain typing data and how useful you find it please write in the box below.

DY: Questions for Physicians

In the questions below, please <u>estimate</u> the number of TB investigations you have been involved in during the last 12 months.

1. In the last year, have you investigated any clusters of 2 or more TB cases because they were contacts of each other (not because of strain type)? *Yes / No*
   If yes, approximately how many investigations were launched based on information gained through contact tracing only?
2. In the last year have you investigated any clusters of 2 or more TB cases because they were linked by strain type along? *Yes / No*
   If yes, approximately how many investigations were launched based on strain type alone?
3. In the last year have you stopped investigating possible transmission because the cases involved had different strain types? *Yes / No*
   If yes, how many incidents does this apply to? Where possible, please provide more information about the incident(s)

End
